# A Molecular Assay to Quantify Male and Female *Plasmodium falciparum* Gametocytes: Results From 2 Randomized Controlled Trials Using Primaquine for Gametocyte Clearance

**DOI:** 10.1093/infdis/jix237

**Published:** 2017-07-06

**Authors:** Will Stone, Patrick Sawa, Kjerstin Lanke, Sanna Rijpma, Robin Oriango, Maureen Nyaurah, Paul Osodo, Victor Osoti, Almahamoudou Mahamar, Halimatou Diawara, Rob Woestenenk, Wouter Graumans, Marga van de Vegte-Bolmer, John Bradley, Ingrid Chen, Joelle Brown, Giulia Siciliano, Pietro Alano, Roly Gosling, Alassane Dicko, Chris Drakeley, Teun Bousema

**Affiliations:** 1 Department of Medical Microbiology, Radboud University Medical Center, Nijmegen, The Netherlands;; 2 Human Health Division, International Centre for Insect Physiology and Ecology, Mbita Point, Kenya;; 3 Malaria Research and Training Centre, Faculty of Pharmacy and Faculty of Medicine and Dentistry, University of Science, Techniques and Technologies of Bamako, Mali;; 4 Department of Laboratory Medicine, Radboud University Medical Center, Nijmegen, The Netherlands;; 5 Medical Research Council Tropical Epidemiology Group, London School of Hygiene and Tropical Medicine, United Kingdom;; 6 Global Health Group, Malaria Elimination Initiative, and; 7 Department of Epidemiology and Biostatistics, University of California, San Francisco;; 8 Dipartimento di Malattie Infettive, Istituto Superiore di Sanità, Rome, Italy; and; 9 Department of Immunology and Infection, London School of Hygiene and Tropical Medicine, United Kingdom

**Keywords:** sex ratio, primaquine, artemisinin, dihydroartemisinin, piperaquine

## Abstract

**Background:**

Single low-dose primaquine (PQ) reduces *Plasmodium falciparum* infectivity before it impacts gametocyte density. Here, we examined the effect of PQ on gametocyte sex ratio as a possible explanation for this early sterilizing effect.

**Methods:**

Quantitative reverse-transcription polymerase chain reaction assays were developed to quantify female gametocytes (targeting *Pfs25* messenger RNA [mRNA]) and male gametocytes (targeting *Pf3D7_1469900* mRNA) in 2 randomized trials in Kenya and Mali, comparing dihydroartemisinin-piperaquine (DP) alone to DP with PQ. Gametocyte sex ratio was examined in relation to time since treatment and infectivity to mosquitoes.

**Results:**

In Kenya, the median proportion of male gametocytes was 0.33 at baseline. Seven days after treatment, gametocyte density was significantly reduced in the DP-PQ arm relative to the DP arm (females: 0.05% [interquartile range {IQR}, 0.0–0.7%] of baseline; males: 3.4% [IQR, 0.4%–32.9%] of baseline; *P* < .001). Twenty-four hours after treatment, gametocyte sex ratio became male-biased and was not significantly different between the DP and DP-PQ groups. In Mali, there was no significant difference in sex ratio between the DP and DP-PQ groups (>0.125 mg/kg) 48 hours after treatment, and gametocyte sex ratio was not associated with mosquito infection rates.

**Conclusions:**

The early sterilizing effects of PQ may not be explained by the preferential clearance of male gametocytes and may be due to an effect on gametocyte fitness.

## Summary

A sensitive molecular assay was developed to quantify male and female *Plasmodium falciparum* gametocytes. Its application in 2 clinical trials demonstrates that the early effects of primaquine may be due to gametocyte fitness rather than sex ratio.

Artemisinin combination therapies (ACTs) have contributed substantially to declines in the burden of falciparum malaria in the last 15 years [1], due to their rapid clearance of asexual stage parasites and activity against immature gametocytes [2, 3]. ACTs reduce the posttreatment transmission potential of parasites from infected humans to mosquitoes more than nonartemisinin treatments [4, 5]. However, because of their incomplete activity against mature gametocytes, patients may remain infective to mosquitoes for up to 2 weeks after ACT treatment [6–8]. The only currently available drug able to clear mature sexual stage malaria parasites is the 8-aminoquinoline primaquine (PQ) [9], which has been used historically for gametocyte clearance as a single dose of 0.75 mg base/kg PQ in combination with a schizonticide [5, 8–12]. Concerns about hemolysis in individuals with glucose 6-dehydrogenase (G6PD) deficiency have contributed to the recent revision of the recommended dose for *Plasmodium falciparum* gametocyte clearance to 0.25 mg base/kg. The World Health Organization recommends that this low dose be provided alongside standard ACT without prior G6PD status screening, for the prevention of *P. falciparum* transmission in areas with ACT-resistant parasites, or areas close to elimination [13].

Though the aim of PQ treatment for *P. falciparum* is a reduction in gametocyte infectivity, there is limited direct evidence on mosquito infection prevalence in individuals treated with the currently recommended low dose [9, 14, 15]. In a recent trial where PQ was combined with a current ACT, the number of individuals infecting mosquitoes dropped from 93.3% to 6.7% over a time window when gametocyte prevalence and density appeared unaffected by PQ [14]. This pattern appears consistent; a review of *P. falciparum* transmission after PQ treatment shows that gametocyte infectivity is generally diminished prior to observable changes in gametocyte abundance [9]. An as yet untested hypothesis is that PQ may disproportionally affect male gametocytes and thus sterilize infections while gametocyte densities, largely determined by the more abundant female gametocytes [16–19], remain stable [20]. In vitro studies with *Plasmodium berghei* show that male gametocytes are more sensitive to a range of antimalarials [21], but PQ requires bioactivation in the liver, so in vitro studies are not possible. Because posttreatment gametocyte densities are commonly below the microscopic threshold for detection, molecular sex-specific gametocyte assays are required to test this hypothesis in clinical trials. The only previously published male gametocyte marker is insufficiently sensitive to quantify low-density male gametocytes [19].

Here, we present results from 2 randomized controlled trials of PQ in combination with dihydroartemisinin-piperaquine (DP), conducted in Kenya and Mali. While this is the first report on the trial in Kenya, gametocyte dynamics and transmission data (but not gametocyte sex ratio) were previously presented for the trial in Mali [14]. In this report, we assess the effect of PQ on gametocyte sex ratio using quantitative reverse- transcription polymerase chain reaction (qRT-PCR) with sex- specific RNA markers [19], including a highly sensitive novel male marker selected using sex-specific *P. falciparum* transcriptomic data (*Pf3D7_1469900*) [22]. Our analysis allowed the first direct assessment of the effect of PQ on gametocyte sex ratio and subsequent infectivity to mosquitoes.

## METHODS

### Study Design and Participants

The Kenyan trial was conducted in Mbita point, western Kenya, between September 2014 and September 2015. The study area is characterized by moderate malaria transmission [23]. During visits to local schools, 5- to 15-year-olds providing written consent for screening were tested for malaria infection by examination of 100 fields of a thick blood smear. Participants providing written informed consent were eligible if they were patent gametocyte carriers (1 gametocyte per 500 white blood cells [WBCs] in a thick film), 5–15 years of age, with *P. falciparum* monoinfection. Exclusion criteria were hemoglobin density of <9.5 g/dL, asexual parasite density of >200000 parasites/µL, body mass index of <16 kg/m^2^ or >32 kg/m^2^, tympanic temperature of >39°C, antimalarial treatment taken within 2 days, recent treatment with drugs known to be metabolized by the cytochrome P450 (CYP) 2D enzyme family, history of adverse reaction to study drugs, blood transfusion within 90 days, history or symptoms of chronic illness, or family history of any condition associated with extended QTc interval. The trial protocol received ethical approval from the Kenya Medical Research Institute Ethics Review Committee (#439), and the London School of Hygiene and Tropical Medicine Observational/Interventional Research Ethics Committee (#7323).

### Procedures

All participants received a 3-day course of DP (Eurartesim, Sigma-Tau, Italy) alone or with a single low dose of PQ (0.25 mg base/kg) on the third day of DP treatment (day 2 of the trial). Enrollees and trial staff other than the trial pharmacist, who was involved only in randomization and drug administration, were blinded to treatment arm allocation.

Primaquine (Sanofi-Aventis US LLC, Bridgewater, New Jersey) was prepared as previously described by dissolving crushed 1-mg tablets into distilled water and mixing with a taste-masking solution to a final concentration of 0.25 mg/kg child weight [8, 24]. Participants assigned to the placebo arm received the same total volume of water and masking solution.

Participants were examined at the study clinic on days 0, 1, 2, 3, 7, and 14 after enrollment. Blood samples were taken at all time points except for day 1, either by fingerpick (day 0, day 2, and day 14) or venipuncture (day 3 and day 7). At all sampling points, hemoglobin levels were quantified by Hemocue photometer (HemoCue AB, Sweden), and asexual parasites and gametocytes were enumerated by examination of a Giemsa-stained thick film counting against 200 or 500 WBCs, respectively, and converted to densities per microliter assuming 8000 WBCs/µL. Two assays were used for gametocyte detection: quantitative nucleic acid–based sequence amplification (QT-NASBA) was used to determine gametocyte prevalence at enrollment and days 2, 3, 7, and 14 following treatment; qRT-PCR was used for gametocyte quantification [25] and sex ratio.

To validate our sex-specific qRT-PCR assays, we first confirmed the ability to generate populations of male and female gametocytes using a recently published PfDynGFP/P47mCherry reporter line (Supplementary Figures 1–3). Then, we developed and optimized sex-specific qRT-PCR assays amplifying messenger RNA (mRNA) specific to the *Pfs25* (female marker) and *Pfs230p* (male marker) genes [19] and more sensitive male targets (Supplementary Table 1). *Pfs230p* also showed limited sensitivity in our preliminary analyses (Supplementary Tables 2 and 3), and in our baseline surveys (gametocytes were undetectable in 9/112 of baseline samples using Pfs230p). We thus designed a new qRT-PCR based on a more abundant gene transcript specific to male gametocytes*; Pf3D7_1469900* (hereafter, *PfMGET* for male gametocyte–enriched transcript) [22]. In the Supplementary Data, we provide details of target selection (Supplementary Table 2), the development and validation of the *PfMGET* qRT-PCR assay (Supplementary Tables 3–6), and details on molecular gametocyte assay methodology for the Kenya and Mali trials. *Pfs25* and *PfMGET* assays showed similar sensitivity, and a threshold for positivity was set at 1 gametocyte per sample (0.002/µL) for both assays.

At day 3 and day 7, blood was provided to mosquitoes for direct membrane feeding assays, as previously described [26]. Low infectivity (38 oocyst-positive mosquitoes out of 8686 dissected, from 2 individuals in the DP arm) prompted additional feeds (n = 32) to be conducted with serum replacement at days 0, 3, and 7. Mosquitoes remained uninfected in these conditions. Later it was discovered that the *Anopheles gambiae* sensu stricto colony was infected with *Microsporidia* species, which have been shown to inhibit *Plasmodium* survival in mosquitoes [27] and precluded meaningful assessments of infectivity in this trial.

To test our findings in an independent dataset, and explore the relationship between sex ratio and infectivity, we performed a subsidiary analysis of blood samples from participants of a single blind, dose-ranging, randomized trial of DP with PQ conducted near Ouelessebougou, Mali [14]. Primaquine was provided with the first dose of DP at baseline, at doses of 0.0625 mg/kg (n = 16), 0.125 mg/kg (n = 16), 0.25 mg/kg (n = 15), and 0.5 mg/kg (n = 17) (control with DP only, n = 16). The main outcomes of this trial were previously reported [14]. For the current study, blood samples were available from baseline and from 2, 3, 7, and 14 days after the administration of PQ. Direct membrane feeding assays for the assessment of infectivity to *A. gambiae* mosquitoes were successfully performed at baseline and at days 2 and 7 [14, 26].

### Sample Size Calculations

Sample size calculations were based on the anticipated primary outcome, the prevalence of infectivity to mosquitoes in membrane feeding assays. A previous study in the same setting showed that 30% of submicroscopic gametocyte carriers and 80% of patent gametocyte carriers infected mosquitoes 7 days after DP treatment [7]. Assuming a conservative estimate of 30% infection after DP and assuming PQ would reduce this to <5% [28], a sample size of 60 participants per treatment arm, allowing for 10% loss to follow-up, was considered sufficient to detect this difference in infection rate with 90% power at a significance level of .05.

### Data Analysis

The primary efficacy endpoint for the Kenyan trial was the mean gametocyte clearance time (ie, number of days until gametocytes become undetectable by QT-NASBA [24] in the DP-PQ arm compared to the DP arm), calculated using previously presented mathematical models that allow clearance time to be extrapolated beyond the period of follow-up [29]. Secondary endpoints were the area under the curve (AUC) of QT-NASBA–based gametocyte density over time (gametocytes/µL^-1^ days) [5] using log_10_ gametocyte density in linear regression models with adjustment for baseline gametocyte density, and qRT-PCR gametocyte prevalence, density, and sex ratio (proportion of total gametocytes that were male) at days 3 and 7. Stata software version 12.0 (StataCorp, College Station, Texas) and SAS software version 9.3 (SAS Institute, Cary, North Carolina) were used for statistical analysis. Baseline measures were compared between treatment arms using Student *t* test, Wilcoxon rank-sum tests, or χ^2^ tests. Differences between treatment arms in gametocyte prevalence and (log_10_-transformed) density after baseline were assessed with linear and logistic models, after adjusting for gametocyte density at baseline. Differences in the proportion of male gametocytes between treatment arms were compared with Wilcoxon rank-sum tests at baseline, and linear models adjusted for baseline gametocyte density after treatment. Proportions within treatment arms were compared to paired baseline measures with Wilcoxon signed-rank test. The accuracy of gametocyte sex ratio estimates depends on the total number of gametocytes detected. To ensure robust sex ratio estimates, ratio analyses were restricted to samples with total qRT-PCR estimated counts of >16 gametocytes per sample [30], resulting in minimum gametocyte per microliter thresholds of 0.032 for Kenyan samples and 0.32 for Malian samples.

## RESULTS

**Figure 1.**
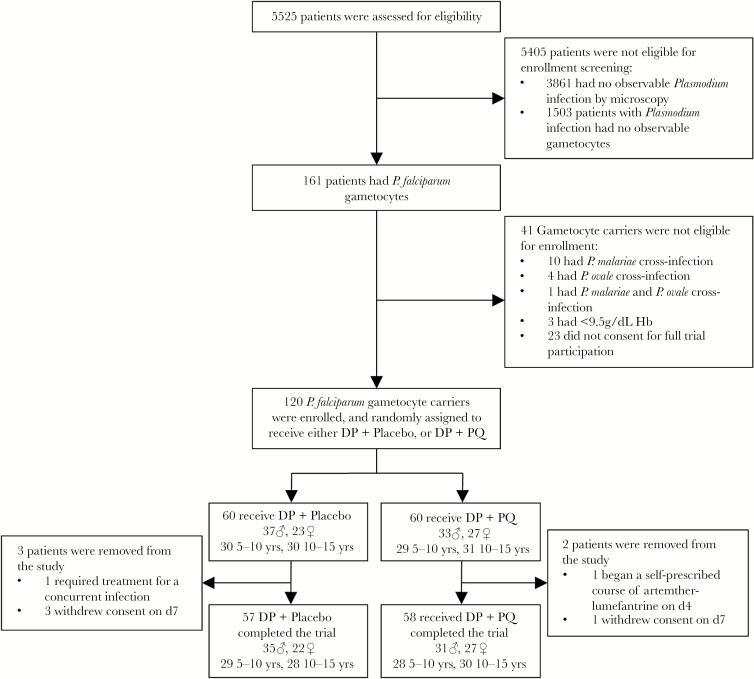
Screening and enrollment criteria. Abbreviations: ♀, female; ♂, male; DP, dihydroartemisinin-piperaquine; Hb, hemoglobin; PQ, primaquine (0.25 mg/kg).

A total of 5525 children were screened, and 120 gametocyte carriers were enrolled into the Kenyan trial (Figure 1). Sixty were assigned to receive DP with a placebo, and 60 were assigned to receive DP-PQ. Four participants in the DP arm and 2 in the DP-PQ arm were lost to follow-up or excluded from the trial. The lowest hemoglobin concentration recorded during the trial was 6.9 g/dL, observed in 1 individual prior to PQ administration who was excluded from the trial. Minimum recorded hemoglobin level in all other participants was 8.6 g/dL.

Gametocyte density at baseline was not significantly different between treatment arms by microscopy (*P* = .705) or qRT-PCR (females: *P* = .692; males: *P* = .784; combined males and females: *P* = .823) (Table 1). The median proportion of male gametocytes at baseline was 0.33 (IQR, 0.22–0.49) in the DP arm, and 0.32 (IQR, 0.17–0.53) in the DP-PQ arm (*P* = .547); that is, a sex ratio of approximately 1 male to 2 females (Table 2).

**Table 1.** Characteristics of the Study Population at Baseline

Characteristic/Measure	DP + Placebo (n = 56)	DP + 0.25 mg/kg PQ (n = 58)	*P* Value
Demographics and health			
Female, % (no./No.)	39.3 (22/56)	46.55 (27/58)	.433
Age, y, median (IQR)	9.5 (7–11)	10 (7–12)	.948
Hemoglobin, g/dL, median (IQR)	11.9 (10.7–13.0)	12.0 (11–12.4)	.753
Microscopy			
Asexual prevalence, % (no./No.)	69.09 (38/55)	59.7 (34/57)	.297
Asexual density, parasites/μL, median (IQR)	360 (0–1600)	120 (0–800)	.133
Gametocyte prevalence, % (no./No.)	100.0 (55/55)	100.0 (56/56)	NC
Gametocyte density, parasites/μL, median (IQR)	32 (16–64)	32 (16–48)	.705
qRT-PCR			
Total gametocyte prevalence, % (no./No.)	100.0 (54/54)	100.0 (57/57)	NC
Total gametocyte density, parasites/μL, median (IQR)	10.84 (3.06–69.97)	11.87 (4.16–55.80)	.823
Male gametocyte prevalence, % (no./No.)	100.0 (54/54)	100.0 (57/57)	NC
Male gametocyte density, parasites/μL, median (IQR)	3.76 (0.78–11.16)	3.09 (0.811–14.14)	.784
Female gametocyte prevalence, % (no./No.)	100.0 (55/55)	100.0 (57/57)	NC
Female gametocyte density, parasites/μL, median (IQR)	6.66 (0.85–36.60)	7.64 (1.59–23.92)	.692

Data are shown as median (IQR) or % (positive/total sample size). Densities are presented for all individuals.

Abbreviations: DP, dihydroartemisinin-piperaquine; IQR, interquartile range; NC, not calculable; PQ, primaquine; qRT-PCR, quantitative reverse-transcription polymerase chain reaction.

**Table 2.** Gametocyte Measures in the Kenyan Study

Measure	DP + Placebo (n = 56)	DP + PQ (n = 58)	*P* Value
Microscopy and QT-NASBA			
Gametocyte prevalence, % (no./No.) (microscopy)			
Day 0	100.0 (55/55)	100.0 (56/56)	NC
Day 2	44.4 (24/54)	40.0 (22/55)	.803
Day 3	44.4 (24/54)	42.9 (24/56)	.811
Day 7	29.6 (16/54)	7.1 (4/56)	.006
Day 14	9.6 (5/52)	0 (0/56)	NC
Gametocyte prevalence, % (no./No.) (QT-NASBA)			
Day 0	98.2 (55/56)	98.3 (57/58)	.980
Day 2	94.6 (53/56)	98.3 (56/57)	.334
Day 3	89.3 (50/56)	96.6 (56/58)	.093
Day 7	85.7 (48/56)	37.9 (22/58)	<.001
Day 14	81.8 (45/55)	8.62 (5/58)	<.001
Gametocyte clearance time, d (95% CI)	49.2 (30.6–67.8)	9.8 (7.7–12.0)	<.001
Gametocyte AUC/ gams/µL^-1^ d, median (IQR)	55.4 (26.9–154.5)	35.4 (12.8–70.1)	.018
Sex-specific qRT-PCR			
Female gametocyte prevalence, % (no./No.) (*Pfs25*)			
Day 0	100.0 (55/55)	100.0 (57/57)	NC
Day 3	92.7 (50/55)	94.7 (51/57)	.816
Day 7	88.9 (48/54)	71.4 (33/56)	.001
Male gametocyte prevalence, % (no./No.) (*PfMGET*)			
Day 0	100.0 (54/54)	100.0 (57/57)	NC
Day 3	96.4 (54/56)	96.5 (55/57)	.931
Day 7	96.3 (52/54)	96.5 (53/57)	.465
Total gametocyte prevalence, % (no./No.) (*Pfs25* and *PfMGET*)			
Day 0	100.0 (54/54)	100.0 (57/57)	NC
Day 3	96.4 (53/55)	96.5 (55/57)	.920
Day 7	96.3 (52/54)	96.4 (52/56)	.933
Female gametocyte density, gams/µL (*Pfs25*)			
Day 0	6.650 (0.851–36.599)	7.640 (1.594–23.916)	.692
Day 3	0.427 (0.054–2.403)	1.307 (0.111–6.277)	.152
Day 7	0.831 (0.080–3.122)	0.003 (0.000–0.055)	<.001
Male gametocyte density, gams/µL (*PfMGET*)			
Day 0	3.762 (0.778–11.164)	3.084 (0.8114–14.142)	.784
Day 3	1.511 (0.303–8.854)	1.920 (0.476–12.387)	.035
Day 7	0.870 (0.213–4.749)	0.137 (0.0140–0.600)	<.001
Total gametocyte density, gams/µL (*Pfs25* and *PfMGET*)			
Day 0	10.836 (3.056–69.973)	11.870 (4.157–55.800)	.823
Day 3	2.864 (0.495–14.707)	4.167 (1.128–18.080)	.030
Day 7	2.509 (0.631–7.136)	0.140 (0.018–0.855)	<.001
Proportion male, median (IQR)			
Day 0	0.338 (0.222–0.493)	0.323 (0.171–0.525)	.457
Day 3	0.692 (0.475–0.910)	0.677 (0.345–0.928)	.634
Day 7	0.457 (0.278–0.732)	0.979 (0.868–1.000)	<.001
Proportion male, median (IQR) (≥16 gams/sample only)			
Day 0	0.332 (0.222–0.492)	0.323 (0.171–0.525)	.457
Day 3	0.700 (0.472–0.911)	0.677 (0.345–0.928)	.635
Day 7	0.441 (0.262–0.683)	0.977 (0.891–0.996)	<.001

Data are presented as median (IQR) or % (positive/total sample size). Densities are presented for all individuals.

Abbreviations: AUC, area under the curve; DP, dihydroartemisinin-piperaquine; gams, gametocytes; IQR, interquartile range; NC, not calculable; PQ, primaquine; qRT-PCR, quantitative reverse-transcription polymerase chain reaction; QT-NASBA, quantitative nucleic acid–based sequence amplification.

### Gametocytocidal Efficacy of Low-Dose PQ

**Figure 2.**
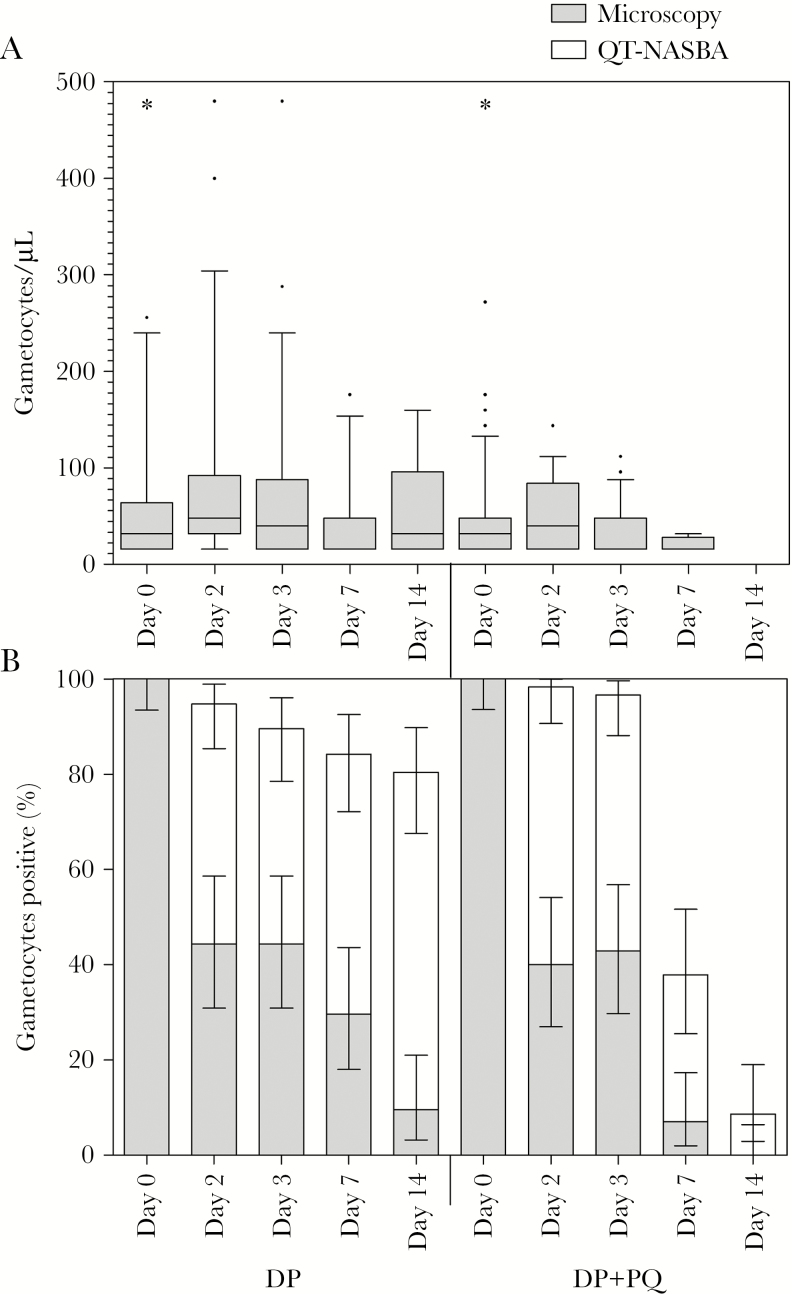
Gametocyte prevalence and density. *A*, Gametocyte density determined by microscopy at all sampling points, presented as the median, interquartile range, and 10th–90th percentiles of gametocytes/µL for gametocyte-positive individuals only. For clarity, 4 data points were not included in the graph: 3 in the dihydroartemisinin-piperaquine (DP)–only arm at day 0 (576, 608, and 686/µL), and 1 in the primaquine (PQ) arm at day 0 (1072/µL). *B*, Gametocyte prevalence determined by microscopy or *Pfs25*-based quantitative nucleic acid–based sequence amplification (QT-NASBA), presented as percentage positive, with 95% confidence interval.

QT-NASBA gametocyte prevalence on day 3 (24 hours after PQ) was 89.3% (50/56) in the DP arm and 96.6% (56/58) in the DP-PQ arm (*P* < .093) (Figure 2; Table 2). At day 7, gametocyte prevalence by QT-NASBA was 85.7% (48/56) in the DP arm and 37.9% (22/58) in the DP-PQ arm (*P* < .001). The estimated mean time to gametocyte clearance in our selective population with high starting gametocyte densities was 49.2 (95% confidence interval [CI], 30.6–67.9) days in the DP arm and 9.8 (95% CI, 7.7–12.0) days in the DP-PQ arm (*P* < .001). For DP, this estimate was based on an extrapolation of QT-NASBA data beyond the duration of follow-up and should therefore be interpreted with caution.

All gametocyte density and sex ratio estimates were based on qRT-PCR. As a consequence of the higher input material for qRT-PCR in the Kenya trial (500 µL of whole blood for extraction, eluted in 10.5 µL water) compared to QT-NASBA (50 µL of whole blood for extraction eluted in 50 µL water), qRT-PCR gametocyte prevalence was considerably higher. At day 3, total gametocyte density determined by qRT-PCR was decreased to 24.81% (IQR, 9.54%–48.29%) of its level at baseline in the DP arm, and 35.73% (IQR, 14.39%–80.82%) in the DP-PQ arm (*P* value after adjustment for baseline density = .03). By day 7, total gametocyte density was decreased to 22.31% (IQR, 7.38%–78.22%) of its baseline level in the DP arm, and 1.43% (IQR, 0.22%–8.19%) in the DP-PQ arm (*P* < .001). Gametocyte AUC was significantly lower in the DP-PQ arm (*P* = .018).

### Effect of DP and PQ on Male and Female Gametocytes

**Figure 3.**
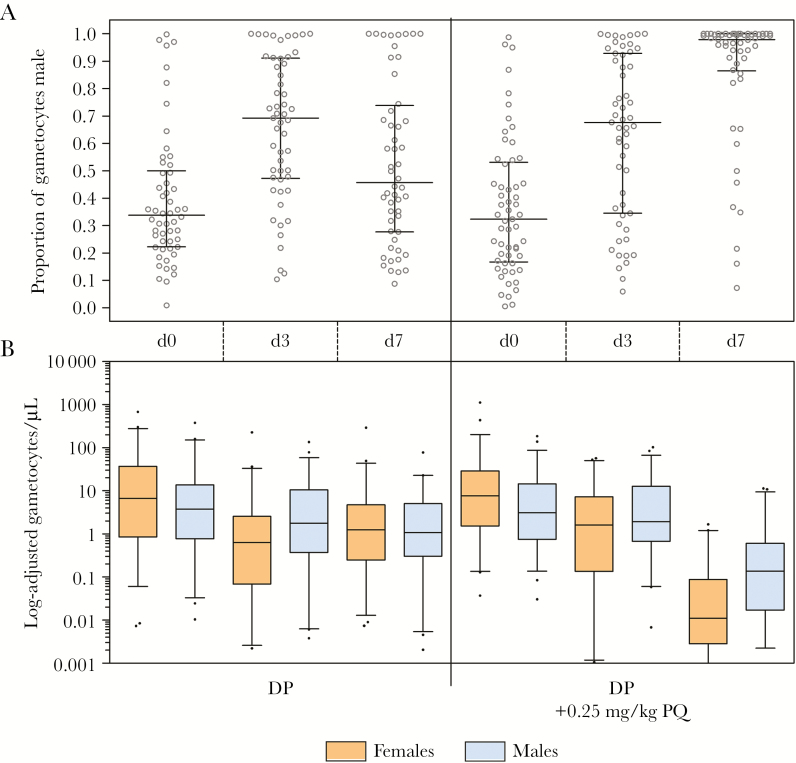
Quantitative reverse-transcription polymerase chain reaction (qRT-PCR)–based male and female gametocyte density and proportion male in the Kenyan study. *A*, Proportion of total gametocyte density that is male (males per µL / [males per µL *plus* females per µL]), presented as individual data points, with the median and interquartile range (IQR). Proportion male was only presented or included in analyses if total gametocyte density was estimated to be ≥16 gametocytes per sample (1 gametocyte/500 µL = 0.032 gametocytes/µL). *B*, Gametocyte density determined by qRT-PCR at baseline (day 0), and 24 (day 3) or 120 (day 7) hours after dihydroartemisinin-piperaquine (DP) or DP plus primaquine (PQ). Female gametocytes were quantified by extrapolating *Pfs25* messenger RNA abundance from standard curves of known quantities of female gametocytes, and vice versa. Density is presented as median, IQR, and 10th–90th percentiles of gametocytes/µL for gametocyte-positive individuals only.

By day 3, female gametocyte density was decreased to a median of 9.1% (IQR, 1.3%–33.5%) of its baseline level in the DP arm, and 14.0% (IQR, 2.9%–65.2%) of its baseline level in the DP-PQ arm (*P* = .152) (Table 2; Figure 3). Male density decreased less substantially over the same period (DP: 40.1% [IQR, 15.8%–86.6%] of baseline; DP-PQ: 61.8% [IQR, 27.7%–191.6%] of baseline; *P* = .035), resulting in male-biased sex ratios in both treatment arms (DP: 0.70 [IQR, 0.47–0.91]; DP-PQ: 0.68 [IQR, 0.35–0.93]; *P* = .625).

At day 7, the density of gametocytes was significantly reduced in the DP-PQ arm relative to the DP arm (females: 0.05% [IQR, 0.0–0.7%] of baseline; males: 3.4% [IQR, 0.4%–32.9%] of baseline; *P* < .001). In the DP arm, sex ratios were lower than at day 3 but still higher than baseline (median proportion male: 0.44 [IQR, 0.26–0.68]; *P* = .002). Sex ratios among the low densities of gametocytes in the DP-PQ arm were significantly more male biased than in the DP arm (median proportion male: 0.98 [IQR, 0.89–1.00]; *P* < .001 for matched measures at baseline, and group comparison with DP arm).

### Effect of DP and PQ on Gametocyte Sex Ratio in Relation to Infectivity to Mosquitoes in an Independent Trial in Mali

**Figure 4.**
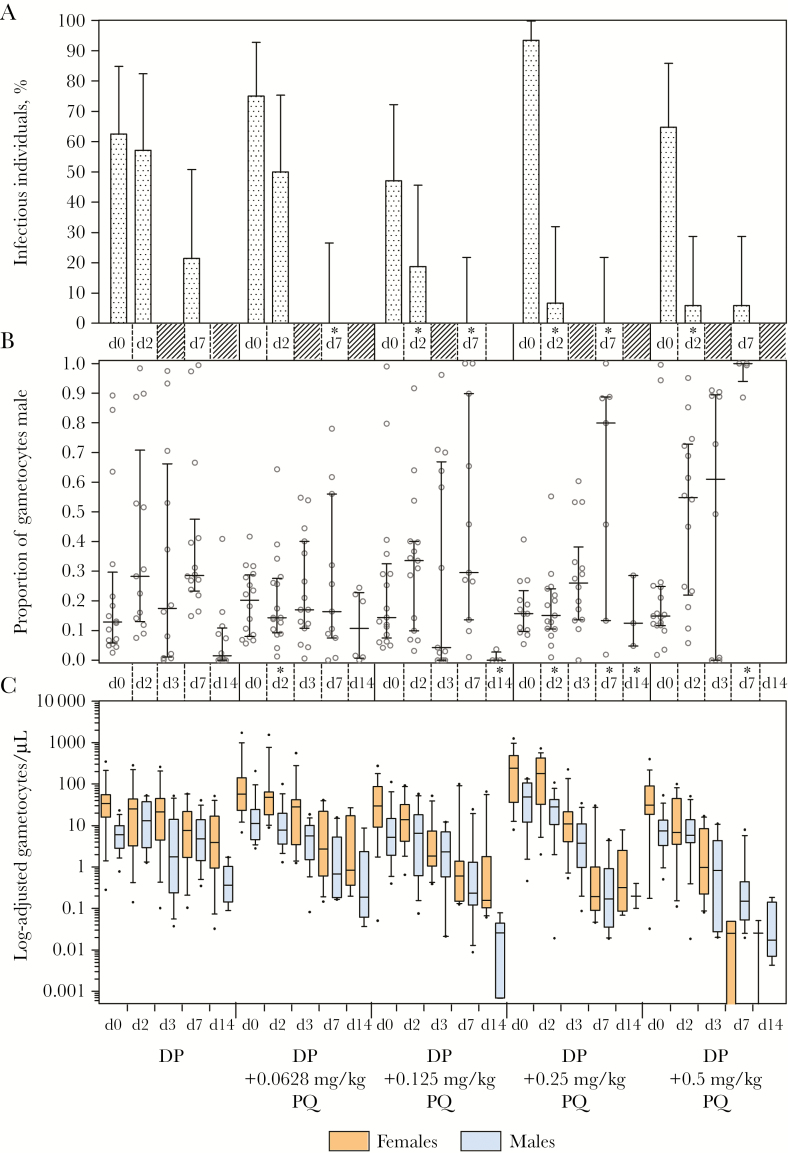
Infectiousness to mosquitoes, quantitative reverse-transcription polymerase chain reaction (qRT-PCR)–based male and female gametocyte density, and proportion male in the Malian study. *A*, Prevalence of infectiousness to mosquitoes among the study population in the direct membrane-feeding assay at days 0, 2, and 7. Asterisks (*) indicate significantly different infectiousness in logistic regressions relative to control. Mosquito infection was determined as the presence of any number of oocysts in the mosquito mid-gut 7 days after feeding. *B*, Proportion of total gametocyte density that is male (males per µL / [males per µL *plus* females per µL]), presented as individual data points, with the median and interquartile range (IQR). Proportion male was only presented or included in analyses if total gametocyte density was estimated to be ≥16 gametocytes per sample (1 gametocyte/50 µL = 0.32 gametocytes/µL). Data from each dose arm and time point are not included in the graph if proportion male was calculable for ≤4 individuals per arm. Asterisks (*) indicate significantly different proportion male in logistic regressions relative to control, adjusted for baseline density. *C*, Gametocyte density determined by qRT-PCR at baseline (day 0), and 24 (day 3) or 120 (day 7) hours after dihydroartemisinin-piperaquine (DP) or DP plus primaquine (PQ). Female gametocytes were quantified by extrapolating *Pfs25* messenger RNA abundance from standard curves of known quantities of female gametocytes, and vice versa. Density is presented as median, IQR, and 10th–90th percentiles of gametocytes/µL for gametocyte-positive individuals only. Median female gametocyte density was calculated from <5 individuals in some dose arms at day 7 (0.5 mg/kg, n = 4) and day 14 (0.5 mg/kg, n = 2). Median male gametocyte density was calculated from <5 individuals in some dose arms at day 14 (0.25 mg/kg, n = 3; 0.5 mg/kg, n = 4).

**Figure 5.**
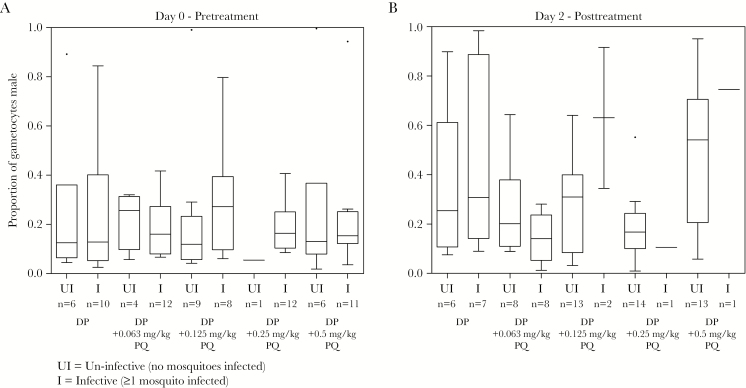
Proportion of male gametocytes in infectious (I) and noninfectious (UI) samples before and after treatment in the Malian study. *A*, Proportions of gametocytes that were male in samples taken at baseline from individuals whose whole blood was infectious or noninfectious to mosquitoes in the direct membrane feeding assay. Mosquito infection was determined as the presence of any number of oocysts in the mosquito mid-gut 7 days after feeding. *B*, Proportions of gametocytes that were male in samples taken at day 2 (48 hours after first dose of dihydroartemisinin-piperaquine [DP] in both the DP and DP plus primaquine [PQ] arms, and only dose of PQ in DP-PQ arms only) from individuals whose whole blood was infectious or noninfectious to mosquitoes in the direct membrane-feeding assay. “n” indicates number of individuals for which proportion male was calculable, and for which mosquito feeding assays were conducted.

*Pfs25* qRT-PCR–based gametocyte prevalence, density, and infectivity to mosquitoes for the trial in Mali have been reported elsewhere [14]. At baseline the median proportion of male gametocytes was 0.15 (IQR, 0.09–0.27) overall, and did not differ between any DP-PQ arms and the DP arm (*P* = .414–.996) (Figure 4). At day 2 (48 hours after PQ administration), there was a significant decrease in the proportion of individuals infecting mosquitoes and the proportion of mosquitoes these individuals infected at all PQ doses of >0.125 mg/kg (Figure 4). The median proportion of gametocytes that were male at day 2 was 0.28 (IQR, 0.14–0.53) in the DP arm. Adjusted for baseline gametocyte density, the proportion of male gametocytes was borderline significantly different in the 0.0625 mg/kg PQ arm (0.14 [IQR, 0.09–0.27]; *P* = .052), but not in any higher-dose PQ arms (proportion male: 0.15–0.55; *P* = .085–.434). The proportion of male gametocytes was similar between DP and DP-PQ arms at day 3 (*P* ≥ .358), but became highly male biased compared to the DP arm in the 0.25 mg/kg and 0.5 mg/kg DP-PQ arms by day 7 (DP: 0.29 [IQR, 0.24–0.41]; DP-PQ 0.25 mg/kg: 0.80 [IQR, 0.13–0.89], *P* = .035; DP-PQ 0.5 mg/kg: 1.0 [IQR, 0.99–1.0], *P* < .001). Increasing PQ doses reduced the female bias of gametocytes by day 7 (Supplementary Table 7). Within each treatment arm, the proportion of male gametocytes at day 7 was significantly higher relative to baseline in the DP (*P* = .016) and the DP-PQ 0.125 mg/kg (*P* = .033) and 0.5 mg/kg (*P* = .043) dose groups, but not in the 0.0625 mg/kg (*P* = .477) or 0.25 mg/kg (*P* = .080) dose groups. There was no significant difference in the proportion of male gametocytes between infectious and noninfectious individuals at baseline (infectious/noninfectious: 55/26; *P* = .964), at day 2 (infectious/noninfectious: 21/57; *P* = .531), or at day 7 (infectious/noninfectious: 5/71; *P* = .244) (Figure 5).

## DISCUSSION

Our findings support the effectiveness of a single dose of PQ (0.25 mg/kg) for shortening gametocyte carriage following ACT treatment [13]. Our sex-specific qRT-PCR suggests that PQ may not preferentially clear male gametocytes. At early time points when PQ has been shown to substantially decrease infectiousness to mosquitoes (24–48 hours) [14, 20], the proportion of gametocytes that were male was comparable between the DP-PQ and DP-only arms.

Before treatment, there is a nonlinear relationship between gametocyte density and infectivity to mosquitoes [31–33]. After PQ treatment, gametocyte density has no obvious relationship with infectivity, and infections appear to be sterilized [14, 20, 24]. White and colleagues highlighted these phenomena in historic studies and urged that trials of PQ efficacy based on gametocyte density measures be interpreted with caution [20]. In a recent PQ efficacy trial, it was postulated that because gametocytes were quantified using *Pfs25*-based molecular assays, only female gametocytes were counted, while infections may have been sterilized by clearance of the smaller undetected male population [14]. If total clearance of one sex was the cause of PQ’s rapid sterilizing effects, a highly sensitive sex-specific assay would be capable of predicting posttreatment infectivity. Though we believe the assay presented in the current manuscript meets these requirements, our data indicate that PQ treatment has a similar effect to ACT treatment in terms of absolute sex ratio, in the days after treatment where infections are sterilized. The extent of the effect varied between the 2 trials we describe in the current study and may relate to the timing of PQ and ACT administration and to the limited sample size of treatment arms in the Malian trial. However, neither trial provides evidence of the hypothesized increase in female bias after PQ treatment; the Kenyan trial indicates that male gametocytes may actually be cleared more slowly than females by both DP and DP-PQ. While gametocyte sex ratio is an important determinant of transmissibility and may be associated with gametocyte density, we observed no relation between posttreatment gametocyte sex ratio and infectivity. Collection of RNA samples from a larger number of gametocyte donors participating in mosquito feeding studies conducted prior to drug administration would allow more rigorous assessment of gametocyte sex ratio and its effect on the likelihood of onward transmission in natural infections.

Our findings do not exclude the possibility that PQ’s sterilizing effects are sex specific. The *P. berghei*–based in vitro dual gamete formation assay has shown that male gametocytes are more susceptible to a range of drugs with different modes of action [21]. The advantage of this system is that its endpoint (exflagellation in males, production of the translationally repressed Pfs25 protein in activated females) is based on gametocytes’ ability to activate (ie, their fitness) rather than the presence of their mRNA, which may remain detectable in nonfunctional intact gametocytes. Quantifying gametocytes based on mRNA transcript numbers has limitations [34]. We used automated RNA extraction to minimize variation in extraction efficiency and assessed mRNA transcript numbers during gametocyte maturation. mRNA transcripts of *Pfs25* and *PfMGET* increased sharply from stage II to stage V gametocytes after which we found no evidence for age-dependent transcription patterns that may explain our findings of a faster reduction of female-specific transcripts following treatment (Supplementary Figure 4). Our findings suggest that the early sterilizing effect of PQ may be a consequence of a reduced fitness, rather than immediate clearance of 1 or both gametocyte sexes. Delves et al showed that percentage inhibition of activation by dihydroartemisinin was approximately 14 times higher for male gametocytes than females [21]. Though this appears to conflict with our observation that male gametocytes are cleared more slowly by DP-PQ, both observations may be valid if (male) gametocytes were rendered nonfunctional by DP-PQ but remained in the circulation during sampling. As such, our study supports the notion that functional assays (be it gametocyte fitness or infectivity) are essential to determine the transmission-blocking properties of antimalarial drugs.

Observations of microscopy’s insensitivity [6, 35–38] and the significance of submicroscopic gametocyte densities for transmission [39–41] have placed great import on quantifying the submicroscopic gametocyte reservoir. The recent realization that *Pfs25* is transcribed specifically, or in far greater abundance in female gametocytes, affects previous interpretations of the *Pfs25* readout as measure of gametocyte density [19], as the male component of gametocyte biomass will have gone largely undetected and thus total gametocyte density will have been underestimated.

The results of the current study shed some light on PQ’s early sterilizing activity. Determining gametocyte sex ratio may have significant utility for examining the relationship between gametocyte density, sex, and infectivity to mosquitoes, and for the assessment of drugs causing clearance of one sex. However, our findings demonstrate that the preferential clearance of one gametocyte sex does not provide an explanation for the rapid sterilizing effect of PQ. Trials assessing transmission-blocking effects of drugs such as PQ should thus continue to rely on functional transmission read-outs such as mosquito feeding assays.

## Supplementary Data

Supplementary materials are available at The Journal of Infectious Diseases online. Consisting of data provided by the authors to benefit the reader, the posted materials are not copyedited and are the sole responsibility of the authors, so questions or comments should be addressed to the corresponding author.

## Supplementary Material

Supplementary_Material

## Notes

***Author contributions.*** T. B., C. D., and P. S. designed the study. W. S., P. S., R. O., M. N., P. O., D. O., S. O., N. M., A. O., V. O., A. M., I. C., J. B., G. S., P. A., R. G., D. E., H. D., M .v. d. V.-B., W. G., and A. D. were involved in data acquisition. S. R., R. W., K. L., and W. S. were involved in the production and validation of the sex-specific gametocyte reporter line. K. L., S. R., and W. S. performed molecular assays to detect and quantify gametocytes. W. S., J. B., and T. B. contributed to statistical analysis. W. S. wrote the first draft of this manuscript. All authors read and approved the final manuscript.

***Acknowledgments.*** We thank the staff of St Jude’s Clinic, at International Centre of Insect Physiology and Ecology, Mbita Point, and particularly David Owaga, Selphas Okach, Nick Makio, George Omweri, Albert Okoth, and Addah Magawa, for their assistance with trial organization, sample collection, and mosquito feeding; *Elke van Veen, for assisting with sample collection and trial management;* Rianne Siebelink-Stoter (Radboudumc), for supporting gametocyte culture and assisting with microscopy; Dr Massimo Sanchez and Dr Valentina Tirelli (Instituto Superiore di Sanita), for assistance in fluorescence activated cell sorting; and Sanofi-Aventis for their kind donation of primaquine.

***Financial support.*** This work was supported by the Bill & Melinda Gates Foundation (AFIRM OPP1034789). T. B. and W. S. are supported by a fellowship from the European Research Council (ERC-2014-StG 639776) and a Marie Curie Career Integration Grant from the European Community’s Seventh Framework Programme (SIGNAL, PCIG-GA-2012–333936).

***Potential conflicts of interest.*** All authors: No reported conflicts of interest. All authors have submitted the ICMJE Form for Disclosure of Potential Conflicts of Interest. Conflicts that the editors consider relevant to the content of the manuscript have been disclosed.
