# Supplementary material for: A Molecular Assay to Quantify Male and Female Plasmodium falciparum Gametocytes: Results From 2 Randomized Controlled Trials Using Primaquine for Gametocyte Clearance
Source: J Infect Dis. 2017 Jul 6;216(4):457–67. doi: 10.1093/infdis/jix237 (PMC5853855; doi:10.1093/infdis/jix237)
Supplement: Supplementary_Material [file jix237_suppl_supplementary_material.docx]

**SUPPLEMENTAL INFORMATION:** A molecular assay to quantify male and female P. falciparum gametocytes: results from two randomised controlled trials using primaquine for gametocyte clearance

**A. Confirmation of sex-specificity of reporter gene expression in the transgenic PfDynGFP/P47mCherry line**

The purity of male and female gametocyte fractions obtained using the PfDynGFP/P47mCherry line was assessed by immune-fluorescence assays (IFA) and microscopy. This line expresses sex-specific fluorescent markers (a chromosomally integrated *GFP* gene under the control of the male specific *Dynein heavy chain* promoter and an *mCherry* gene in an episomal plasmid, whose expression is controlled by the female specific *P47* promoter), which allow separation of male and female gametocytes by fluorescence-activated cell sorting (FACS) [1]. A detailed description of the development of the reporter line, culture conditions and sorting into sex specific populations by flow cytometry has been reported [1]. For the current experiments, conducted at Istituto Superiore di Sanità in Rome, Italy, sorting was performed based on GFP signal only. On day 5 following NAG addition, 4x10^7^ stage III gametocytes were purified by MACS column, sorted based on GFP expression and fractions of GFP+ (male gametocytes) and GFP- (female gametocytes) were brought back into separate cultures. On day 12, sorted populations had progressed to stage V of gametocyte maturation and were analysed by IFA using the gametocyte specific anti-Pfg27 rat antibody [2] and the female gametocyte specific anti-Pfg377 rabbit antibody [3], generated against the recombinant B region of Pfg377 [4], and by microscopy examination of Giemsa stained smears. IFA analysis showed that 100% of the gametocytes in the GFP+ fraction was Pfg27+/Pfg377- (Figure S1, Figure S2) as expected for male gametocytes. This was confirmed by examination of the Giemsa stained smears showing that gametocytes exhibit the diagnostic cytoplasmic pink staining (Figure S3).

This independently confirmed previous observations [1] that the portion of GFP+ gametocytes which also show a weak expression of the mCherry reporter (Figure S1A) are in fact male gametocytes. By contrast, gametocytes in the GFP- fraction were 83% Pfg27+/Pfg377+, and thus female gametocytes (Figure S1, S2). Examination of the Giemsa stained smears confirmed that the vast majority of these gametocytes exhibit the diagnostic cytoplasmic blue staining (Figure S3). A minor fraction the GFP- gametocytes (17%) were Pfg27+/Pfg377-. These could be male gametocytes from the population of non-fluorescent gametocytes. These results fully support the conclusion that mCherry+/GFP- gametocytes are female gametocytes.

**Figure S1. Counts of live fluorescent gametocytes in the GFP+ and GFP- populations and summary of IFA results**

| 1. Counts of live fluorescent gametocytes in GFP+ and GFP- populations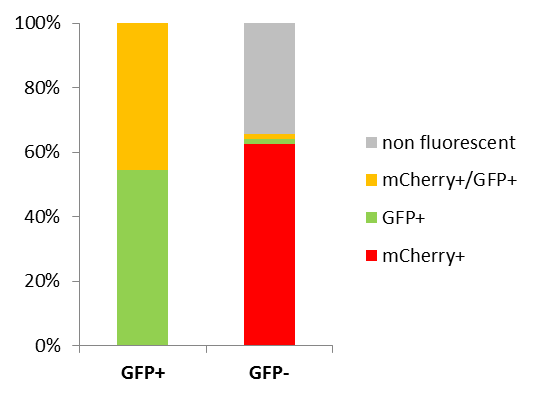 | 1. Double IFA with α-Pfg377 and α-Pfg27 antibodies on acetone fixed gametocyte smears of the GFP+ and GFP- populations.   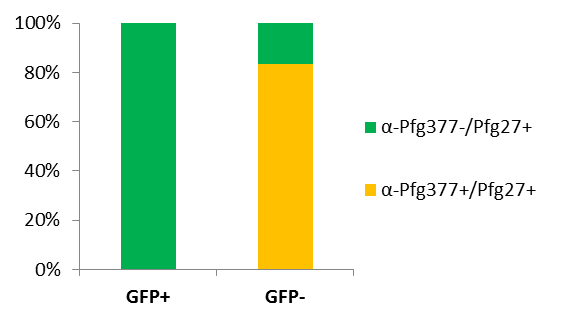 |
| --- | --- |

**Figure S2. Representative IFA results**

**GFP+ gametocytes** (100% Pfg27+/Pfg377-)

**
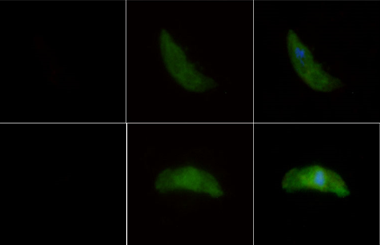
**

**α-Pfg377 α-Pfg27 merge + Hoechst**

**GFP- gametocytes** (83% Pfg27+/Pfg377+)

**
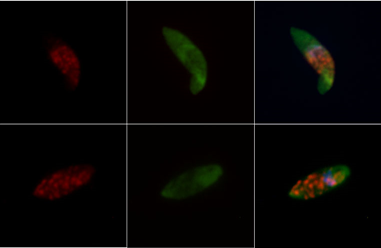
**

**α-Pfg377 α-Pfg27 merge + Hoechst**

**Figure S3. Giemsa staining of sorted GFP+ and GFP- stage V gametocyte populations.** For slide preparation, purified gametocytes were mixed with blood for the purpose of smearing.

**
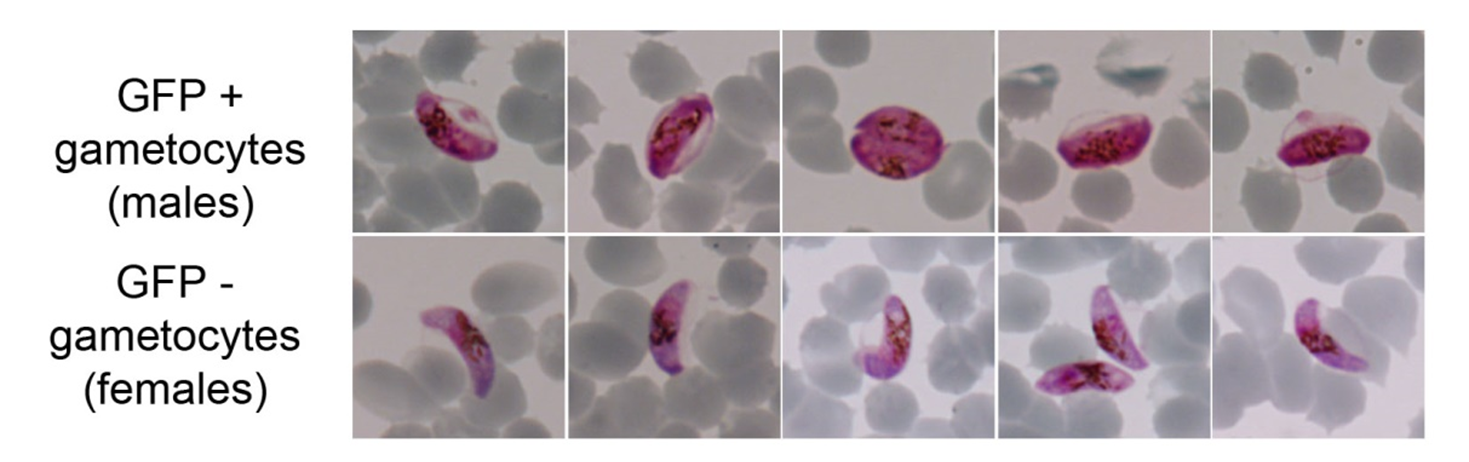
**

**B. Sex specific qRT-PCR development**

***Target selection***

Sex-specific qRT-PCR assays using primers based on *Pfs25* (female marker; *PF3D7_1031000*) and *Pfs230p* (male marker; *PF3D7_0208900*) were previously reported [5]. We tested a novel male marker, *Pf3D7_1469900* (*PfMGET*), based on a recently published sex-partitioned transcriptomic analysis of *P. falciparum* gametocytes [1]. We selected this gene based on sex-specificity, asexual stage over gametocyte ratio, and qRT-PCR performance. **Table S1** shows the 10 genes most abundantly transcribed in male gametocytes, their abundance in female gametocytes, and their ratio in females to males. Furthermore, publically available illumina based mRNA sequence libraries (www.plasmodb.org) from seven *P. falciparum* life stages (ring, early trophozoite, late trophozoite, schizont, gametocyte stage II, gametocyte stage V, and ookinete) indicates a ratio of transcript abundance in asexual stages over mature gametocytes (in fragments per kilobase per million mapped reads (FPKM); average of all asexual stages/gametocyte stage V) of 0.36 for Pfs230p and 0.07 for *PfMGET* [6]. Proteins aligning to *PfMGET* were not observed in a recent proteomic analysis of sex separated stage V *P. falciparum* gametocytes (expressing GFP under the control of the male specific *α-tubulin II* promoter), and were detected at very low level in previous proteomic analyses [1, 7]. *PfMGET* was selected for further testing based on evidence of low transcription in asexual stage parasites, high transcript abundance in gametocytes, male gametocyte specificity, and on its adaptability to diagnostic qRT-PCR; *PfMGET* contains introns, which allowed the design of intron spanning primers (**Table S2**), thereby obviating the necessity for DNase treatment that may reduce sensitivity [8].

**Table S1. Transcript levels of top 10 male genes.** Data are from *Table S2 in Lasonder et al. 2016*. Values for MG (male gametocytes) and FG (female gametocytes) are normalised reads per kilobase per million reads [RPKM]. Ratio is FG RPKM/MG RPKM.

| ***Gene ID*** | ***Old Gene ID*** | **Product** | **MG** | **FG** | **RATIO FG/MG** |
| --- | --- | --- | --- | --- | --- |
| *PF3D7_1201600* | *PFL0080c* | NIMA related kinase 3 (NEK3) | 17813.52 | 236.63 | 0.01 |
| ***Pf3D7_1469900 (PfMGET)*** | ***PF14_0667*** | **conserved Plasmodium protein, unknown function** | **10110.85** | **208.93** | **0.02** |
| *PF3D7_0406200* | *PFD0310w* | sexual stage-specific protein precursor (Pfs16) | 7991.31 | 10195.62 | 1.28 |
| *PF3D7_1413200* | *PF14_0131* | conserved Plasmodium protein, unknown function | 3859.07 | 362.29 | 0.09 |
| *PF3D7_0422300* | *PFD1050w* | alpha tubulin 2 | 3618.96 | 89.36 | 0.02 |
| *PF3D7_1311100* | *PF13_0060* | meiosis-specific nuclear structural protein 1, putative | 2548.67 | 12.59 | 0.00 |
| *PF3D7_1105100* | *PF11_0062* | histone H2B (H2B) | 2426.04 | 658.45 | 0.27 |
| *PF3D7_0617800* | *PFF0860c* | histone H2A (H2A) | 2111.91 | 2573.03 | 1.22 |
| *PF3D7_0726000* |  | 28S ribosomal RNA | 1616.88 | 1465.62 | 0.91 |
| *PF3D7_0630800* | *PFF1495w* | conserved Plasmodium protein, unknown function | 1541.28 | 235.37 | 0.15 |

**Table S2. Primer sequences for the Pfs25 female marker, and male markers Pfs230P and PfMGET.**

| **Gene target** | **Forward primer** | **Reverse primer** |
| --- | --- | --- |
| *Pfs25* [9] | GAAATCCCGTTTCATACGCTTG | AGTTTTAACAGGATTGCTTGTATCTAA |
| *Pfs230P* [5] | CCCAACTAATCGAAGGGATGAA | AGTACGTTTAGGAGCATTTTTTGGTAA |
| *PFMGET* | CGGTCCAAATATAAAATCCTG | GTGTTTTTAATGCTGGAGCTG |

For preliminary testing of the new marker, qRT-PCR assays were performed using standard curves of mixed sex NF54 gametocyte mRNA (ten-fold serial dilutions from 10^6^ to 10 gametocytes/mL). Briefly, NF54 *P. falciparum* was cultured in the semi-automated culture system and started at 5% hematocrit and 0.5% parasitemia [10, 11]. Treatment with N-acetyl-glucosamine on day 7 eliminated asexual parasites, and mature stage IV/V gametocytes were harvested after 13–15 days, which was confirmed by Giemsa stained thin blood films. Gametocyte samples were serially diluted into 50µL blood, and stored in 250µL RNAprotect lysis buffer (Qiagen, Hilden, Germany). Total nucleic acid was extracted using a MagNAPure LC automatic extractor (Total Nucleic Acid Isolation Kit–High Performance; Roche Applied Science, Indianapolis, IN, USA) followed by RQ1 DNaseI digest (Promega, Sunnyvale, CA, USA) and cDNA synthesis (High Capacity cDNA Reverse Transcription Kit, Applied Biosystems, Foster City, CA, USA). DNaseI digestion was performed for all mixed sex gametocyte samples. qRT-PCR was performed as described previously [12], except that 1µL cDNA was used in the 20µL final reaction mix, and primer concentration was reduced to 225nM.

Despite a probable loss of sensitivity incurred to the *PfMGE*T assay through DNaseI digestion, *PfMGET* mRNA appeared more abundant than *Pfs230p* mRNA, reflecting evidence of the transcripts relative abundance *per capita* [1, 6]. Data from assays conducted in parallel on the same material are shown in **table S3**. The lower CTs for the Pfs25 may represent higher transcript copy numbers per parasite, but are also likely the result of female bias in the mixed sex gametocyte culture.

**Table S3. *Pfs25*, *Pfs230p*, and *PFMGET* qRT-PCR, with standard curves of mixed sex NF54 gametocytes.** Data in the table come from assays performed on the same batch of gametocyte material. The data represent means of triplicate qRT-PCR CT values, except for Pfs230 which was performed once using this material.

| **Parasite/stage** | **Quantity (gams/mL)** | ***Pfs25*** | ***Pfs230p*** | ***PfMGET*** |
| --- | --- | --- | --- | --- |
| **Gametocytes (NF54)**  *Mixed sex & NAG synchronised* | **10^6** | 20.72 | 27.2 | 24.34 |
|  | **10^5** | 23.83 | 29.84 | 27.03 |
|  | **10^4** | 26.94 | 32.19 | 30.48 |
|  | **10^3** | 30.14 | N/A | 32.82 |
|  | **10^2** | 34.56 | N/A | 33.74 |
|  | **10^1** | 34.17 | N/A | N/A |

*PfMGET = PF3D7_1469900*

NAG = N-acetyl-glucosamine

Gams/mL = Gametocytes per mL

***Gametocyte specificity***

To empirically assess the specificity of *PfMGET* mRNA to gametocytes, and determine expression at different asexual stages, qRT-PCR assays were performed using standard curves of synchronised NF54 asexual mRNA. Asexual parasites were synchronized by the selection of late trophozoites and schizonts on a 63% percoll density gradient, followed by a 5% sorbitol treatment, killing the remaining schizonts after 5 hours, ensuring tight synchronization of parasites within a 5 hour window. Ring stage parasites were harvested 10, 20, 30 and 40 hours after percoll synchronization respectively. Parasite samples were lysed in L6 buffer and extracted immediately or frozen at -80^o^C until further use. Extraction and PCR conditions were the same as for the unsynchronised gametocyte samples, except that cDNA input to qRT-PCR was 2 µL.

Though input to PCR was double for the Pfs230p and PFMGET assays compared to Pfs25, transcript became undetectable at similar levels in the Pfs25 and male marker assays (threshold for detection generally >10^4) (**Table S4**).

**Table S4.** **Standard curves of synchronised asexual stage NF54 *P. falciparum*.** Data in the table are qRT-PCR CT values.

| **Parasite/stage** | **Quantity (gams/mL)** | ***Pfs25*** | ***Pfs230p*** | ***PfMGET*** |
| --- | --- | --- | --- | --- |
| **Asexuals 10hr** | **10^6** | 32.18 | 32.54 | 32.66 |
|  | **10^5** | 34.91 | N/A | N/A |
|  | **10^4** | N/A | N/A | N/A |
| **Asexuals 20hr** | **10^6** | 35.91 | N/A | 32.47 |
|  | **10^5** | N/A | 37.46 | 34.83 |
|  | **10^4** | N/A | N/A | N/A |
| **Asexuals 30hr** | **10^6** | 31.65 | 32.50 | 32.51 |
|  | **10^5** | 33.51 | 32.89 | 34.23 |
|  | **10^4** | N/A | N/A | N/A |
| **Asexuals 40hr** | **10^6** | 30.04 | 31.89 | 32.45 |
|  | **10^5** | 34.78 | N/A | N/A |
|  | **10^4** | N/A | N/A | N/A |

*PfMGET = PF3D7_1469900*

N/A = mRNA not detectable

Gams/mL = Gametocytes per mL

***Male/female gametocyte specificity/sensitivity***

We then used sorted male and female gametocytes to assess specificity of our assay. For this standard curves of purified female and male gametocytes (ten-fold serial dilutions from 10^6^ to 10 gametocytes/mL) were constructed from gametocyte mRNA of the transgenic parasite line PfDynGFP/P47mCherry. [1]. Briefly, NF54 WT and PfDynGFP/P47mCherry parasites were cultured in the semi-automated culture system and started at 5% hematocrit and 0.5% parasitaemia [10, 11]. Treatment with N-acetyl-glucosamine (NAG) on day 7 eliminated asexual parasites to harvest mature stage IV/V gametocyte after 13–15 days, confirmed by Giemsa stained thin blood films. Gametocytes were concentrated in 37°C culture medium, separated from uninfected erythrocytes using a 63% Percoll density gradient, and subsequently taken up in a 4°C suspended activation (SA) buffer (10 mM Tris, pH 7.3, 170 mM NaCl, 10 mM glucose). Using a FACS AriaII SORP (BD) gametocytes were first separated from uninfected red blood cells using forward and sideward scatters, and then were sorted based on signal intensity of the fluorescent proteins (green fluorescent protein (GFP) or mCherry). A recent proteomic analysis of a *P. falciparum* gametocyte strain expressing GFP under the control of the male specific *α-tubulin II* promoter confirms the male specificity of Dynein heavy chain protein expression, but indicates shared expression of P47 in male and female gametocytes [13]..In the current study, pure male and female specific populations were obtained by excluding fluorescence negative and double positive cells. Gametocytes were FACS-sorted to obtain populations of GFP+ mCherry- (male gametocytes) and GFP- mCherry+ (female gametocytes) gametocytes, which were kept at 4°C in suspended activation (SA) buffer. Assessed by fluorescence microscopy, no GFP positive parasites were observed in the mCherry+ populations, and no mCherry positive parasites were observed in the GFP+ populations (data not shown). FACS purity analysis indicated the single fluorescent cell populations were isolated with 99.7% purity. Preliminary testing was conducted with 100 µL standard curves (**table S5**).

**Table S5. qRT-PCR with *Pfs25* and *PfMGET* and purified male and female gametocyte populations.** Data in the table are qRT-PCR CT values, for three biological replicates of purified male and female gametocyte material. Extraction procedures were as for the mixed sex preliminary samples, except that sample input to the magNAPure extractor was 100µL and elution was in 100µL of milliQ water.

| **Quantity (gams/mL)** | **Batch 1** |  | **Batch 2** |  | **Batch 3** |  |  |  |
| --- | --- | --- | --- | --- | --- | --- | --- | --- |
|  | ***PfMGET*** | ***Pfs25*** | ***PfMGET*** | ***Pfs25*** | ***PfMGET*** | ***Pfs25*** | ***PfMGET*** | ***Pfs25*** |
| **10^6** | 19.55 | 20.42 | 19.13 | 21.47 | 19.89 | 20.91 | 27.25 | 25.17 |
| **10^5** | 23.04 | 23.56 | 22.24 | 24.23 | 23.05 | 24.14 | 30.57 | 27.78 |
| **10^4** | 26.38 | 27.12 | 25.72 | 27.72 | 26.98 | 28.15 | N/A | 31.55 |
| **10^3** | 29.47 | 29.58 | 28.50 | 31.79 | 30.92 | 31.61 | N/A | N/A |
| **10^2** | 33.52 | 33.33 | 32.84 | 32.39 | 33.34 | 35.29 | N/A | N/A |
| **10^1** | N/A | 34.86 | N/A | N/A | 34.74 | N/A | N/A | N/A |

| Males **(GFP+; mCherry-)** |
| --- |
| Females **(GFP-; mCherry+)** |
|  |

*PfMGET = PF3D7_1469900*

N/A = mRNA not detectable

Gams/mL = Gametocytes per mL

Sex-specific gametocyte standard curves were constructed utilising the separate GFP+, mCherry- and GFP-, mCherry+ gametocyte populations. Standard curve volume in blood, storage, and extraction procedure matched those of clinical trial samples in the current study. Briefly, mRNA was extracted and eluted in 10.5µL of water using ZR Whole Blood RNA miniprep kit (Zymo Research, Irvine, CA) from blood stored at a ratio of 1 part to 3 in Blood RNA buffer (Zymo Research, Irvine, CA) at -20 °C. cDNA was synthesised (High Capacity cDNA Reverse Transcription Kit, Applied Biosystems) directly from nucleic acids for the *PfMGET* assay, and after DNase treatment (RQ1 DNaseI, Promega) for the *Pfs25* and *Pfs230p* assays. The *PfMGET* assay was also performed using the DNAse digested RNA, for comparison. qRT-PCR reactions were conducted as previously described [9], except that primer concentrations were reduced to 225 nM and cDNA input into the final 20 µL reaction mixture was adjusted to 2 µL. The results of testing using the optimised standard curves is shown in **Table S6.**

**Table S6. qRT-PCR with *Pfs25*, *Pfs230p* and *PfMGET* primers using male (GFP+) and female (mCherry+) high volume gametocyte standard curves.** Data in the table are single qRT-PCR CT values. Sample input was 500µL. Total NA = Not DNAseI treated, RNA = DNAseI treated. Bold = Standard curve and sample preparation for analysis of Kenyan samples (for Malian samples, same preparation but lower volume – 50µL to match the sample volume).

|  | **Extraction material** | **Total NA** | **RNA** | | | **RNA** | | |
| --- | --- | --- | --- | --- | --- | --- | --- | --- |
|  | **Marker** | ***PfMGET*** | | ***P230p*** | ***Pfs25*** | ***PfMGET*** | ***P230p*** | ***Pfs25*** |
| **Quantity (gams/mL)** | **10^6** | 14.43 | **16.64** | 19.35 | 22.05 | 24.02 | 25.89 | **15.53** |
|  | **10^5** | 17.83 | **19.69** | 22.39 | 25.57 | 27.63 | 29.19 | **20.02** |
|  | **10^4** | 21.44 | **23.16** | 25.99 | 29.27 | 30.37 | 32.94 | **23.13** |
|  | **10^3** | 24.98 | **26.18** | 29.06 | 30.75 | N/A | N/A | **26.26** |
|  | **10^2** | 28.21 | **29.29** | 33.1 | N/A | N/A | N/A | **30.08** |
|  | **10^1** | 30.98 | **33.44** | N/A | N/A | N/A | N/A | **33.44** |

| Males **(GFP+; mCherry-)** |
| --- |
| Females **(GFP-; mCherry+)** |
|  |

*PfMGET = PF3D7_1469900*

N/A = mRNA not detectable

Gams/mL = Gametocytes per mL

Removing the DNAse digestion step from RNA preparation for the *PfMGET* assay improved the assays sensitivity notably (increase of approximately 2 CT’s in all biological replicates). The Pfs25 and *PfMGET* assays detection limits for gametocytes of their cognate sex were <0.01 gametocytes/µL (the lowest gametocyte density used in the standard curves), while the detection limit of the Pfs230p assay for male gametocytes was closer to 0.1 gametocytes/µL. When the female and male markers were tested using standard curve material from the alternate sex, the sensitivity of the assays decreased by a factor of 100-1000 (10^3/10^4 male gametocytes were detectable using the Pfs25 assay, 10^1 were detectable using the *PfMGET* assay, and vice versa for female parasites). The residual Pfs25 and 146 transcript detected in standard curve of the opposite sex indicates either that both genes are transcribed in small quantities in gametocytes of the opposite sex, or that the purified standard curves were contaminated by approximately 0.1-1% of the opposite sex (in line with the purity of the sorting as assessed by FACS purity analysis). As the level of background signal was similar for both Pfs25 and 146, it was deemed unlikely to have an effect on the quantification of either sex or the determination of sex ratio in mixed sex samples. Sex ratio analyses were restricted to samples with total qRT-PCR estimated counts of >16 gametocytes per sample (gametocyte/µL thresholds of 0.032 for Kenyan samples, and 0.32 for Malian samples), to improve reliability [14].

For all clinical trial samples, separate aliquots of mRNA were processed for the Pfs25/Pfs230p assays and for the *PfMGET* assay, as for the standard curve material. Only samples and standard curves material retained for the Pfs25/Pfs230p assays were DNAse digested. qRT-PCR quantification was performed with matching sex standard curves to ensure that male/female specific RNA transcript abundance in field samples could be reliably converted to counts of male or female gametocytes. For the Pfs25 and *PfMGET* assays a cut off for positivity was set as 1 gametocyte per sample (2 gametocytes per mL/0.002 gametocytes per µL).

**C. *Pfs25* and *PfMGET* transcript abundance during gametocyte maturation**

Relating gametocyte transcript numbers to gametocyte densities comes with uncertainties. One of these uncertainties is related to the stability of the number of transcripts during gametocyte maturation. Figure S5 presents mRNA transcript counts for *Pfs25* and *PF_14699000* (*PfMGET*) in a time-course experiment. Culturing of NF54 was done without prior gametocyte sex-sorting and female (top panel) and male (bottom panel) transcripts were quantified in the same source culture material using Illumina RNAseq with paired reads, 37bp in length. Transcript counts after normalization for library size are presented for d4 till d16 after synchronization; stage IV gametocytes were observed on day 5 and stage V gametocytes on day 7. These results indicate that for both male and female markers transcript abundance is low in stage IIb gametocytes and peaks in stage V gametocytes after which transcript numbers appear stable (*Pfs25*) or may decline at late time-points (*PfMGET*).

**Figure S4. Transcript stability during gametocyte maturation.**


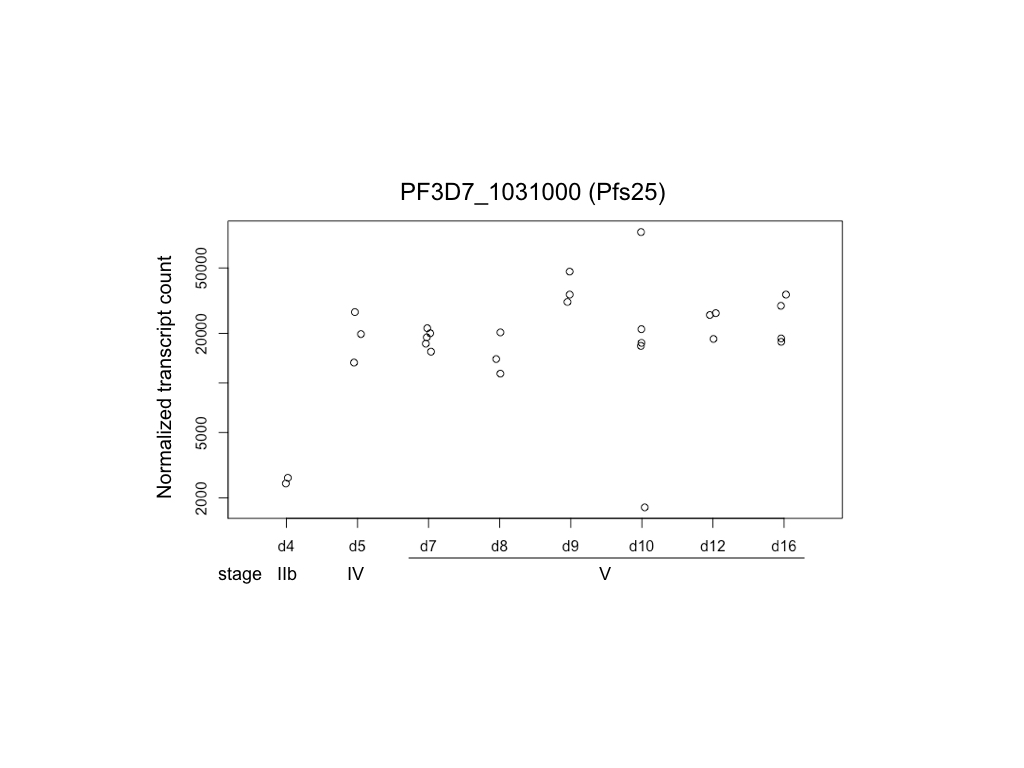


|  |
| --- |
| 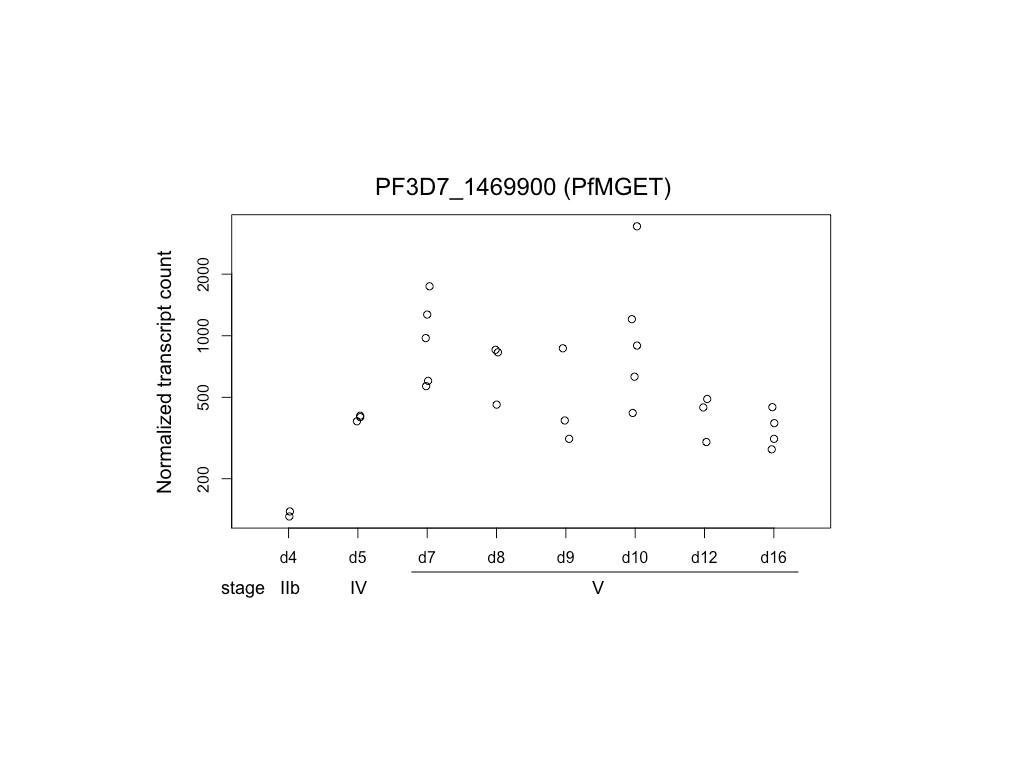 |

**D. Additional clinical trial methodology**

**Details of molecular assays on the samples from clinical trials in Kenya and Mali.**

**Kenya.** Two assays were used for gametocyte detection: quantitative nucleic acid-based sequence amplification (QT-NASBA) was used to determine gametocyte prevalence at enrolment and d2, d3, d7 and d14 following treatment; quantitative reverse-transcriptase PCR (qRT-PCR) was used for gametocyte quantification [12].

QT-NASBA was performed following MagNAPure LC automatic extraction (Total Nucleic Acid Isolation Kit – High Performance, Roche Applied Science) from 50 µL blood stored in 250 µL of RNA protect cell reagent (Qiagen, Germany) and using previously described protocols and a KCl concentration of 50mM in the final reaction mix [15], giving detection limits of approximately 0.02-0.1 gametocytes/µL [15, 16]. A positivity cut-off was set at 1 gametocyte per sample (0.02/µL).

All qRT-PCR in the Kenya trial used high volume blood samples taken at days 0 (250 µL), 3 (500 µL) and 7 (500 µL). mRNA was extracted and eluted in 10.5 µL of water using Zymo RNA extraction kits from blood stored in Zymo lysis buffer (Zymo Research, Irvine, CA). cDNA was synthesised directly from nucleic acids for the male *PfMGET* assay, for which the primers are intron-spanning, and after DNase treatment (RQ1 DNase I Digest Kit, Promega) for the female *Pfs25* and male *Pfs230p* assays, using High Capacity cDNA Reverse Transcription Kits (Applied Biosystems, Foster City, CA). qRT-PCR reactions used previously described protocols [5, 17], except that primer concentrations were reduced to 225 nM and cDNA input into the final 20 µL reaction mixture was adjusted to 2 µL. To convert qRT-PCR results to male or female gametocyte density, standard curves (ten-fold serial dilutions from 10^6^ to 10 gametocytes/ml) of separate male and female gametocyte populations were isolated by fluorescence activated cell sorting using a transgenic parasite line expressing male and female specific fluorescence markers [1], as described above. Male gametocytes in trial samples were quantified against male gametocyte standard curves (prepared from total nucleic acids), both using the *PfMGET* qRT-PCR. Female gametocytes in trial samples were quantified against DNAseI treat female gametocyte standard curves, both using the *Pfs25* qRT-PCR. *Pfs25* and *PfMGET* assays showed similar sensitivity, and a threshold for positivity was set at 1 gametocyte per sample (0.002/µL) for both assays.

**Mali.** For qRT-PCR in the Mali trial, 50 µL blood samples stored in L6 buffer (Severn Biotech, Kidderminster, UK) were used for the sex-specific qRT-PCR assays, which were performed under the same conditions as the Kenyan samples except that cDNA input to PCRs was 1 µL.

**E. Additional clinical trial data**

**Table S7. Proportion of male gametocytes in Mali by day and treatment arm.**

| **Day** | **DP alone** | **DP+0.0625mg/kg PQ** | | **DP+0.125mg/kg PQ** | | **DP+0.25mg/kg PQ** | | **DP+0.5mg/kg PQ** | | **Dose trend** |
| --- | --- | --- | --- | --- | --- | --- | --- | --- | --- | --- |
|  | **Prop. Male (Median/IQR)** | **Prop. Male (Median/IQR)** | **P value (Coeff., SE)** | **Prop. Male (Median/IQR)** | **P value (Coeff., SE)** | **Prop. Male (Median/IQR)** | **P value (Coeff., SE)** | **Prop. Male (Median/IQR)** | **P value (Coeff., SE)** | **P value (Coeff., SE)** |
| **0** | 0.128  (0.061-0.269) | 0.202  (0.082-0.285) | 0.543  (-0.05,0.081) | 0.144  (0.086-0.291) | 0.878  (0.012,0.08) | 0.157  (0.101-0.203) | 0.414  (-0.071,0.086) | 0.149  (0.117-0.247) | 0.996  (0.000,0.08) | 0.927  (0.013,0.142) |
| **2** | 0.283  (0.141-0.529) | 0.143  (0.096-0.271) | 0.052  (-0.179,0.09) | 0.335  (0.099-0.401) | 0.434  (-0.071,0.091) | 0.150  (0.105-0.241) | 0.085  (-0.174,0.099) | 0.548  (0.233-0.722) | 0.291  (0.098,0.092) | 0.063  (0.327,0.173) |
| **3** | 0.174  (0.014-0.618) | 0.170  (0.108-0.400) | 0.516  (-0.08,0.122) | 0.042  (0.000-0.638) | 0.849  (-0.024,0.124) | 0.261  (0.137-0.331) | 0.761  (-0.041,0.135) | 0.610  (0.000-0.891) | 0.254  (0.153,0.133) | 0.135  (0.358,0.236) |
| **7** | 0.285  (0.238-0.411) | 0.164  (0.075-0.560) | 0.586  (-0.066,0.12) | 0.295  (0.135-0.895) | 0.599  (0.061,0.114) | 0.800  (0.133-0.887) | 0.035  (0.35,0.161) | 1.000  (0.994-1.000) | <0.001  (0.571,0.148) | <0.001  (1.308,0.282) |
| **14** | 0.014  (0.000-0.102) | 0.107  (0.010-0.222) | 0.272  (0.065,0.057) | 0.000  (0.000-0.018) | 0.322  (-0.064,0.063) | 0.125  (0.048-0.285) | 0.128  (0.126,0.079) |  |  | 0.927  (0.013,0.142) |

Prop. Male = Proportion of gametocytes male

Dose trend = A trend in the proportion of gametocytes that were male with increasing PQ dose was tested using a linear regression model where the DP-only arm had a value of 0 and the other arms had the value of the PQ dose (e.g. 0.0625, 0.125, 0.25, 0.5).

**References**

1. Lasonder E, Rijpma SR, van Schaijk Ben CL, et al. Integrated transcriptomic and proteomic analyses of P. falciparum gametocytes: molecular insight into sex-specific processes and translational repression. Nucleic Acids Research **2016**.

2. Olivieri A, Camarda G, Bertuccini L, et al. The Plasmodium falciparum protein Pfg27 is dispensable for gametocyte and gamete production, but contributes to cell integrity during gametocytogenesis. Molecular microbiology **2009**; 73:180-93.

3. Suarez-Cortes P, Sharma V, Bertuccini L, et al. Comparative Proteomics and Functional Analysis Reveal a Role of Plasmodium falciparum Osmiophilic Bodies in Malaria Parasite Transmission. Molecular & cellular proteomics : MCP **2016**; 15:3243-55.

4. Alano P, Read D, Bruce M, et al. COS cell expression cloning of Pfg377, a Plasmodium falciparum gametocyte antigen associated with osmiophilic bodies. Mol Biochem Parasitol **1995**; 74:143-56.

5. Schneider P, Reece SE, van Schaijk BCL, et al. Quantification of female and male Plasmodium falciparum gametocytes by reverse transcriptase quantitative PCR. Mol Biochem Parasitol **2015**; 199:29-33.

6. López-Barragán MJ, Lemieux J, Quiñones M, et al. Directional gene expression and antisense transcripts in sexual and asexual stages of Plasmodium falciparum. BMC Genomics **2011**; 12:587.

7. Silvestrini F, Lasonder E, Olivieri A, et al. Protein Export Marks the Early Phase of Gametocytogenesis of the Human Malaria Parasite Plasmodium falciparum. Molecular & Cellular Proteomics **2010**; 9:1437-48.

8. Wampfler R, Timinao L, Beck H-P, et al. Novel Genotyping Tools for Investigating Transmission Dynamics of Plasmodium falciparum. The Journal of Infectious Diseases **2014**; 210:1188-97.

9. Wampfler R, Mwingira F, Javati S, et al. Strategies for Detection of *Plasmodium* species Gametocytes. PLoS ONE **2013**; 8:e76316.

10. Ponnudurai T, Lensen AH, Leeuwenberg AD, Meuwissen JH. Cultivation of fertile Plasmodium falciparum gametocytes in semi-automated systems. 1. Static cultures. Transactions of the Royal Society of Tropical Medicine and Hygiene **1982**; 76:812-8.

11. Ponnudurai T, Lensen AH, Meis JF, Meuwissen JH. Synchronization of Plasmodium falciparum gametocytes using an automated suspension culture system. Parasitology **1986**; 93 ( Pt 2):263-74.

12. Pett H, Gonçalves BP, Dicko A, et al. Comparison of molecular quantification of Plasmodium falciparum gametocytes by Pfs25 qRT-PCR and QT-NASBA in relation to mosquito infectivity. Malaria Journal **2016**; 15:539.

13. Miao J, Chen Z, Wang Z, et al. Sex-Specific Biology of the Human Malaria Parasite Revealed from the Proteomes of Mature Male and Female Gametocytes. Molecular & Cellular Proteomics **2017**.

14. Robert V, Read A, Essong J, et al. Effect of gametocyte sex ratio on infectivity of Plasmodium falciparum to Anopheles gambiae. Transactions of the Royal Society of Tropical Medicine and Hygiene **1996**; 90:621-4.

15. Schneider P, Schoone G, Schallig H, et al. Quantification of Plasmodium falciparum gametocytes in differential stages of development by quantitative nucleic acid sequence-based amplification. Molecular and Biochemical Parasitology **2004**; 137:35-41.

16. Schneider P, Bousema T, Omar S, et al. (Sub)microscopic Plasmodium falciparum gametocytaemia in Kenyan children after treatment with sulphadoxine-pyrimethamine monotherapy or in combination with artesunate. International Journal for Parasitology **2006**; 36:403-8.

17. Dicko A, Brown JM, Diawara H, et al. Primaquine to reduce transmission of Plasmodium falciparum malaria in Mali: a single-blind, dose-ranging, adaptive randomised phase 2 trial. The Lancet Infectious Diseases **2016**.
